# CCL19 enhances CD8^+^ T-cell responses and accelerates HBV clearance

**DOI:** 10.1007/s00535-021-01799-8

**Published:** 2021-07-03

**Authors:** Yan Yan, Wei Zhao, Wei Liu, Yan Li, Xu Wang, Jingna Xun, Chantsalmaa Davgadorj

**Affiliations:** 1 Laboratory for Infection and Immunity, The Fifth People’s Hospital of Wuxi, Affiliated Hospital of Jiangnan University, Wuxi, China; 2 Hepatology Institute of Wuxi, The Fifth People’s Hospital of Wuxi, Affiliated Hospital of Jiangnan University, Wuxi, China; 3 The State Key Laboratory of Pharmaceutical Biotechnology, Chemistry and Biomedicine Innovation Center, Model Animal Research Center, Medical School of Nanjing University, Nanjing, 210061 China; 4 Shanghai Public Health Clinical Center, Fudan University, Shanghai, China

**Keywords:** Chemokine (C–C motif) ligand 19 (CCL19), Hepatitis B virus, CD8^+^ T cell, Apoptosis, Regulatory T cell (T_reg_ cell)

## Abstract

**Background:**

Chemokine (C–C motif) ligand 19 (CCL19) is a leukocyte chemoattractant that plays a crucial role in cell trafficking and leukocyte activation. Dysfunctional CD8^+^ T cells play a crucial role in persistent HBV infection. However, whether HBV can be cleared by CCL19-activated immunity remains unclear.

**Methods:**

We assessed the effects of CCL19 on the activation of PBMCs in patients with HBV infection. We also examined how CCL19 influences HBV clearance and modulates HBV-responsive T cells in a mouse model of chronic hepatitis B (CHB). In addition, C–C chemokine-receptor type 7 (CCR7) knockdown mice were used to elucidate the underlying mechanism of CCL19/CCR7 axis-induced immune activation.

**Results:**

From in vitro experiments, we found that CCL19 enhanced the frequencies of Ag-responsive IFN-γ^+^ CD8^+^ T cells from patients by approximately twofold, while CCR7 knockdown (LV-shCCR7) and LY294002 partially suppressed IFN-γ secretion. In mice, CCL19 overexpression led to rapid clearance of intrahepatic HBV likely through increased intrahepatic CD8^+^ T-cell proportion, decreased frequency of PD-1^+^ CD8^+^ T cells in blood and compromised suppression of hepatic APCs, with lymphocytes producing a significantly high level of Ag-responsive TNF-α and IFN-γ from CD8^+^ T cells. In both CCL19 over expressing and CCR7 knockdown (AAV-shCCR7) CHB mice, the frequency of CD8^+^ T-cell activation-induced cell death (AICD) increased, and a high level of Ag-responsive TNF-α and low levels of CD8^+^ regulatory T (T_reg_) cells were observed.

**Conclusions:**

Findings in this study provide insights into how CCL19/CCR7 axis modulates the host immune system, which may promote the development of immunotherapeutic strategies for HBV treatment by overcoming T-cell tolerance.

**Supplementary Information:**

The online version contains supplementary material available at 10.1007/s00535-021-01799-8.

## Introduction

Approximately 250 million people worldwide are chronically infected with hepatitis B virus (HBV) and are at high risk of developing liver steatosis, cirrhosis, end-stage liver failure or hepatocellular carcinoma [1, 2]. Currently approved therapeutic strategies, including interferon-alpha (IFN-α), nucleoside and nucleotide analogs only provide limited efficacy in sustaining long-term HBV “eradication” [1, 3, 4], and the occurrence of adverse events leads to treatment termination or poor adherence [5].

In primary HBV infection, the virus is completely eradicated in approximately 90% of adults, with the remaining individuals developing viral persistence and disease progression. Immune defense against HBV infection is complex, with the mechanisms having attracted considerable attention which may represent a promising approach to combat chronic hepatitis B (CHB). HBV-responsive CD8^+^ T-cell responses play a crucial role in viral clearance at onset of infection, while these responses become rather weak later on during chronic infection, which is called CD8^+^ T-cell exhaustion [2, 6, 7].

In our previous study concerning innate immunity, factors such as Toll-like receptors (TLRs) were identified as the first line of defense in HBV removal by initiating intracellular signaling pathways to produce interferons (IFNs) and other cytokines, which induce an HBV-responsive immune response [8]. Chemokines are also known to serve as an important link between innate and adaptive immunity and can activate the innate immune response [9, 10]. Chemokine (C–C motif) ligand 19 (CCL19) is an immunological homeostasis chemokine that orchestrates CCR7-expressing inflammatory T-cell recruitment and the homeostatic trafficking of lymphocytes and dendritic cells along a chemokine gradient [10, 11], and it also takes part in enhancing T-cell responses and antitumor immunity [12]. CCL19 is the only chemokine known to effectively stimulate β-arrestin-mediated CCR7 phosphorylation and internalization, which results in receptor desensitization and antigen (Ag)-presenting DC migration [13], thereby affecting T-cell responses. The chemokine-receptor CCR7 is widely expressed by leukocytes, including mature DC, B, naïve T, Th1, and Th2 cells as well regulatory T cells (T_reg_ cells). During HBV infection, the HBx protein takes part in the evasion of antiviral immunity by mimicking an autophagy receptor-like molecule and downregulating signal protein expression. Furthermore, HBx also functions downstream of the chemokine-receptor axis, limiting the apoptotic response and resulting in chronic HBV infection [14]. Studies on CCR7 signaling and its influence on viral pathogenesis have elucidated the complex interplay among viruses, target cells and host immune responses, especially with respect to how changes in the number of CCR7-positive cells during chronic infection and latency promote viral invasion, e.g., HIV-1 [15–17], influenza virus [18, 19], dengue virus (DENV) [20–22], respiratory syncytial virus (RSV) [23–25], etc. On the other hand, viral proteins can act as antagonists or inappropriate agonists to use chemokine receptors as modes of cellular invasion [11, 14, 26], such as HIV-1 [27]. Thus, reducing CCR7 during viral infection may inhibit viral invasion, but whether this mechanism is a practical approach to prevent HBV invasion requires further study.

Chemokines can direct leukocytes to migrate to tissues, especially the hepatic vascular bed in the liver, a unique low-flow environment in which the leukocytes are recruited through infiltration during homeostatic immune surveillance and in response to infection or injury [28]. Hepatic nonparenchymal liver cells (NPCs) have a potential role in suppressing T-cell activation [29]. Given that T-cell immunity can be directly modulated by CCL19 or indirectly regulated by antigen-presenting cells (APCs), such as plasmacytoid dendritic cells, in the present study, we attempted to systematically analyze the mechanism of action of CCL19 in HBV clearance using a mouse model. Understanding the underlying mechanism of HBV clearance directed by CCL19 may contribute to the development of useful therapeutic options. In the present study, we examined the effect of CCL19, a potent agonist for CCR7, on immune activation and assessed its role in promoting HBV clearance in mice with chronic HBV infection.

## Materials and methods

### Patient samples and IFN-γ induction

Peripheral blood mononuclear cells (PBMCs) from HBV-infected patients (HBV DNA was positive in serum) and healthy individuals (as assessed by physical examination) as healthy controls (HCs, not infected with HBV) were obtained from the in- and out-patient clinic of Wuxi Infectious Disease Hospital, Affiliated Hospital of Jiangnan University, from January 2019 to February 2020. The present study was approved by the Institutional Review Board of Hospital Ethics Committee (Ethics No. 2020-014-1).

HBV-infected patients included one was acute hepatitis B (AHB), 14 individuals were CHB, one individual was hepatocellular carcinoma (HCC) and one individual was HCs. The AHB patient was recently infected by treatment for cavities (Supp. Table 1). Some of these chronic hepatitis B patients did not develop glutamate transaminase (ALT) elevated. The simultaneous elevation of the level of ALT and the copies of HBV DNA represents developing HBV-associated hepatitis. The CHB individuals were diagnosed as patients with a previous history of a clinical course of HBV infection or were HBsAg positive for over 6 months and remained positive for HBsAg and/or HBV DNA (25). Hepatocellular carcinoma patients were all transformed from CHB.

PBMCs were obtained and stimulated with or without 100 ng/ml of recombinant human CCL19/MIP-3β protein (R&D systems, China) or were stimulated with a peptide of the hepatitis B core (HBc) 93–100 epitope (MGLKFRQL) (Proimmune Company, the United Kingdom), the HBc + CCL19, HBc + CCL19 + shCCR7 (2 × 10^8^ TU/ml) or CCR7 mAb (3D12, Abnova, Taiwan), the HBc + CCL19 + phosphatidylinositol 3-kinase phosphoinositide 3-kinase (PI3K) pharmacological inhibitor LY294002 (Absin Bioscience, China), and the PBMCs of experimental groups were activated by anti-human CD3 (1 μg/ml) and anti-human CD28 (1 μg/ml) (eBioscience, USA).

### Plasmids and shCCR7 constructs

The plasmid pAAV/HBV1.2 was kindly provided by Prof. Chen Pei-Jer (Graduate Institute of Clinical Medicine, College of Medicine, National Taiwan University, Taiwan), which contains 1.2-fold of the full-length DNA HBV genome [30]. A murine CCL19 plasmid construct was described previously [31]. A lentiviral vector (LV) receptor CCR7 small interfering RNA construct (LV-shCCR7) and an adeno-associated virus (AAV) vector CCR7 small interfering RNA construct (AAV-shCCR7) were used for CCR7 downregulation in human lymphocytes and in the mouse model, respectively. In vitro, LV-shCCR7 transfection of human PBMCs was performed using Lipofectamine 6000 (Thermo Fisher, USA) following the manufacturer’s instructions. In vivo, hydrodynamic injection (H.I.) of AAV-shCCR7 into mouse tail veins was followed by CCR7 knockdown at 7 days post injection. The CCR7 shRNA sequences were as follows [32]: 5′-GCG UCA ACC CUU UCU UGU ATT-3′ (sense) and 5′-GAC GCA GUU GGG AAA GAA CAU-3′ (antisense), and the constructs were designed and generated by Shanghai Sangon Biotech Company (China).

### Mouse model

Male C57BL/6 mice (Model Animal Research Center, Collaborative Innovation Center of Genetics and Development, Nanjing University) aged 6–8-weeks were bred and maintained under specific pathogen-free (SPF) conditions in the Model Animal Research Center, Nanjing University. CHB mouse model were transfected with pAAV/HBV1.2 by H.I. in a volume of phosphate-buffered saline (PBS) equivalent to 10% of their body weights (i.e. 10 μg/2 ml) within 5–8 s [33, 34]. For the CCL19 over expressed mouse model, mice were transfected with a murine CCL19-ecoding plasmid by H.I (10 μg). For the CCR7 knockdown mouse model, mice were transfected with 1.92 × 10^10^ vg/ml per mouse commercialized shCCR7 (AAV-shCCR7) and AAV-negative control (NC) (Sangon Biotech, China) as a control by H.I. 1 week before the establishment of CHB mouse model. At the same time, naïve mice were only injected with murine CCL19-ecoding plasmid and AAV-shCCR7 as control groups. All experimental procedures involving mice were approved by the Institutional Animal Care and Use Committee of the Model Animal Research Center (MARC) and Nanjing Biomedical Research Institute of Nanjing University (NBRI) and performed according to the guidelines of the Jiangsu Laboratory Animal Science Association (Nanjing, China, Approval ID: LY-01).

### Sampling and hepatic Ag-presenting cell (APC) isolation

Blood from C57BL/6 mice was sampled at 2 and 4 weeks post infection (wpi), and serum and peripheral blood lymphocytes (PBMCs) were isolated and detected. Sera were analyzed for HBsAg, HBeAg, HBs antibodies (HBsAb), HBV DNA and CCL19 mRNA expression at interval week after H.I.

Human and murine PBMCs from whole blood treated with anticoagulant were isolated with lymphocyte separation medium (Dakewe, China) following the manufacturer’s instructions, and red blood cells were lysed with Lysing Buffer (BD Biosciences, USA). Single cell suspensions of murine splenocytes were isolated from mouse spleens under sterile conditions according to a previously described protocol [35]. To isolate hepatic APCs, the mouse livers were separated under sterile conditions at 60 days post infection (dpi), and primary murine hepatocytes and NPCs, including Kupffer cells (KCs), liver sinusoidal endothelial cells (LSECs), NK cells and other immature myeloid-derived cells (iMDCs) recruited from the blood (CD11b^+^) were isolated and cultured as described previously [36, 37]. Briefly, the livers of sacrificed mice were immediately perfused with 15 ml of PBS preheated to 37 °C and subsequently digested into homogenized cells with an enzyme solution containing 0.05% collagenase type II (Sigma-Aldrich, Germany). iMDCs were purified from NPCs using a BD Cell Separation Magnet, F4/80^+^ or CD146^+^ microbeads and magnetic activated cell separation (MACS) solution (Thermo Fisher, USA) sorting according to the manufacturer’s instructions, to obtain KCs and LSECs, respectively. Liver tissue samples were preserved in 10% formaldehyde for subsequent immunohistochemical (IHC) analysis.

All mouse experiments were performed according to the guidelines established by the Institutional Animal Care and Use Committee at Model Animal Research Center, Collaborative Innovation Center of Genetics and Development, Nanjing University.

### CCL19 over expression in vivo and in vitro

In vivo, the ability of CCL19 over expression to induce NPCs or other leucocyte subclasses activation, and the expansion of KCs was assessed by flow cytometry and performed according to the subclass analysis of murine PBMCs at 4 wpi (middle period of viral clearance or chronic infection). In vitro, the ability of CCL19 to stimulate hepatocytes and iMDCs and indirectly induce lymphocyte activation and secretion of cytokines was assessed by flow cytometry at 60 dpi (late period of viral clearance or chronic infection) and performed according to lymphocyte surface and intracellular staining as described previously [29, 34]. In brief, C57BL/6 mice were pretreated (H.I.) with the murine-encoding CCL19 plasmid and HBV plus CCL19, and the composition of murine NPCs was analyzed at 60 dpi. That is, isolated hepatocytes (6 × 10^5^ per well), LSECs (4 × 10^5^ per well) and KCs (2 × 10^5^ per well) from each group were seeded in 24-, 48- and 96-well plates, respectively, precultured for 24 h, and stimulated with 100 ng/ml recombinant murine CCL19 protein (PeproTech, USA). After 24 h, the supernatants of prestimulated cells were removed, washed two times with PBS and then co-cultured with freshly isolated splenocytes from respective experimental mice, and co-stimulated with anti-mouse CD3 (1 μg/ml) and anti-mouse CD28 (1 μg/ml) antibodies (Abs) (eBioscience, USA) for 48 h at a ratio of 1:2 (iMDCs:splenocytes). The cytokines in the supernatants or cells were detected by flow cytometry assay. The supernatants were filtrate through 0.45 μm membrane (Merck Millipore, USA). Cytokines secretion from the inner cells was stopped by adding 5 μg/ml brefeldin A (eBioscience, USA) for 4 h before intracellular staining.

### Chemotaxis assay

Chemotaxis assays for clinical patient PBMCs were conducted as previously described [35]. Using a microchamber transwell system with a 3-μm-pore-size (Corning Costar, USA), PBMCs (2 × 10^6^ per well) were suspended in 300 μl of RPMI 1640 medium supplemented with 0.5% fetal bovine serum (FBS) and then transferred to the upper chambers of transwells in triplicate. Then, 600 μl of RPMI 1640 medium with or without (negative control) 50 ng/ml recombinant human CCL19 protein (PeproTech, USA) medium was added to the bottom chambers. Subsequently, the plates were incubated for 90 min in an incubator at 37 °C under an atmosphere with 5% CO_2_, and the number of migrated cells in the lower chambers was counted. The rates of migration were graphed as the fold change and migrated cells assayed in triplicate. Data from triplicate samples were analyzed.

### Flow cytometry

All antibodies used for flow cytometry were purchased from BD biosciences and eBioscience. Cells were examined by flow cytometry with a BD FACSAria™ III flow cytometer (BD Biosciences) and analyzed with FlowJo 11.0. Cell surface and intracellular markers for iMDCs were stained for flow cytometry analysis as previously described [29]. Briefly, KCs (F4/80^+^, CD11b^+^) and DCs (CD11b^+^, CD11c^+^, CD8^−^ and CD4^−^) were identified, and cell debris and dead cells were excluded from the analysis based on scatter signals and staining with E506. After 48 h coculture, the cytokine concentrations in the supernatant of each group of PBMCs were assessed using a BD Cytometric Bead Array (CBA) Mouse Th1/Th2 Cytokine kit (BD Biosciences, USA). Surface and intracellular staining of human PBMCs was performed with anti-human monoclonal antibodies (mAbs) against the following proteins: CD3, CD19, CD45, CCR7, CD127, CD25, FoxP3, PD-1 (CD279), TNF-α, IFN-γ and IL-2 fluorescent Abs, and the dye 7AAD were used (BD Biosciences, USA). Murine PBMCs were classified and analyzed based on leucocyte surface markers by flow cytometry as the following cell types: CCR7^+^, CD279^+^ (PD-1^+^), T cell (CD3^+^, CD45^+^, CD4^+^ and CD8^+^), NK cell (NK1.1^+^), B cell marker (CD19^+^), DC (CD45^−^,CD11c^+^, and CD86^+^), macrophages (CD45^−^, IA/IE^+^, and F4/80^+^), monocytes (CD45^−^, IA/IE^−^, and Ly6C^+^) and granulocytes (CD45^+^, CD11b^+^, and Ly6G^+^). Cell classification was also performed based on staining with the dye E506 and the detection of intracellular cytokines, using TNF-α, IFN-γ and IL-2 fluorescent Abs.

### Apoptotic cell analysis

Isolated splenocytes from each mouse models were stimulated with HBc peptide for 48 h and then stained with an Annexin V-FITC/PI Apoptosis kit (Yeasen Biotech, China) following the manufacturer’s instructions. Early (Annexin V^+^ and PI^−^) and late (Annexin V^+^ and PI^+^) apoptotic CD8^+^ T cells were detected by flow cytometry.

### ELISA, detection of HBV replication and CCL19 transcription

After H.I., the mice were regularly bled to monitor the serum levels of hepatitis B surface antigen (HBsAg), hepatitis B e antigen (HBeAg), hepatitis B surface antibody (anti-HBs), HBV DNA and CCL19 mRNA at weekly intervals [33]. In brief, serum levels of HBsAg, HBeAg and HBsAb were detected with ELISA according to the reagent instructions (Jingmei Biotech, China). The levels of nucleocapsid HBV DNA, CCL19 and a housekeeping RNA gene extracted from 60 mg of mouse liver were detected on an ABI PRISM 7500 Sequence Detection System (ABI PRISM™, China) by real-time RT-PCR with 2 × SYBR Green PCR Master Mix (Sangon Biotech, China). Total DNA and RNA were purified from serum using an All-In-One DNA/RNA/Protein Mini-preps kit (Sangon Biotech, China). HBV DNA detection was performed using *care*HBV PCR Assay V3 reagents (QIAGEN, China) according to the manufacturer’s instructions. cDNA was generated for mouse CCL19 and the housekeeping gene β-actin by reverse transcription AMV First Strand cDNA Synthesis kit (Sangon Biotech, China) following the manufacturer’s instructions. Real-time RT-PCR primers for murine CCL19 and the internal control housekeeping gene β-actin were designed as follows: CCL19, 5′-CTG CCT GTC TGT GAC CCA GCG CCC C-3′ (sense) and 5′-ACT TCT TCA GTC TTC GGA TGA TGC G-3′ (antisense); and β-actin, 5′-GCG AGC ACA GAG CCT CGC CTT TG-3′ (sense) and 5′-GAT GCC GTG CTC GAT GGG GTA C-3′ (antisense). Fold changes in CCL19 mRNA expression in mucosa were determined and graphed using the 2^−ΔΔCt^ method.

### Immunohistochemistry (IHC) analysis

Liver tissues were collected from mice at 4 wpi. HBc Ag in murine liver was visualized by IHC staining using mouse anti-HBc Abs [14E11] (Abcam, USA). The tissues were also stained with hematoxylin. The expression levels of HBc Ag were defined using a quick score, which was calculated according to a previously described method [38].

### Statistical analysis

Statistical analysis comparing two groups were performed using the nonparametric or Student’s *t* test, while ANOVA was used when more than two groups were compared. Statistical analyses were performed using GraphPad Prism 8, and the data are presented as the means ± standard errors of the mean (SEMs). *p* values < 0.05 were considered significant.

## Results

### CCL19 increases the frequency of Ag-responsive IFN-γ-expression in HBV-infected patients

It is generally acknowledged that robust, polyclonal, and viral-specific CD4^+^ and CD8^+^ T-cell responses and neutralized antibody responses are major determinants of the resolution of HBV infection [39, 40]. Antiviral function can be mediated by IFN-γ and TNF-α secreted by CD8^+^ T cells or by Ag-nonspecific macrophages [41, 42]. T-cell-derived IFN-γ-regulated genes can contribute to regulate an adaptive T-cell response that inhibits viral replication and kills infected cells, thereby terminating the infection [42]. Since CCL19 appears to trigger APCs and T-cell trafficking, we evaluated its possible roles in enhancing CD8^+^ T-cell function in HBV-infected patients and elucidated its possible functions and mechanisms in promoting HBV clearance. Our results showed that CCL19-mediated HBc primary and restimulated PBMCs to produce higher level of IFN-γ in supernatants, whereas this enhanced effect was partially decreased by CCR7 knockdown and CCR7 monoclonal Ab (mAb) even though adding CCL19 protein and completely abrogated by PI3K signal inhibitor LY294002 (Supp. Fig. 1a, c). Notably, AHB, HCC, and CHB individuals’ PBMCs produced higher levels of IFN-γ after CCL19 stimulation, compared with negative control (NC, without CCL19 and HBc stimulation), while produced poor secretion of IFN-γ after HBc stimulation, and the role of CCL19 is also weakened (Supp. Fig. 1b). In contrast, CCL19 stimulation enhanced the frequencies of Ag-responsive IFN-γ-expressing CD8^+^ T cells by approximately twofold but did not significantly alter the frequencies of CCR7 expression in PBMCs from CHB patients, (NC vs + CCL19 groups; + HBc vs + HBc + CCL19 groups) (Supp. Fig. 1d). Similarly, freshly isolated PBMCs from CHB (with HBV infection) and HC (without HBV infection) individuals were stimulated with HBc peptide, CCL19 and LY294002 in vitro for 72 h and then assessed for their ability to migrate to CCL19. As shown in Supp. Fig. 1e, for lymphocytes stimulated with HBc plus CCL19 peptide or protein produced ~ 1.5-fold more (median) PBMCs migrating in response to CCL19 compared with HBc stimulation alone in hepatitis B patients (HBV +). Taken together, these results indicate that CCL19 is capable of promoting the Ag-responsive CCR7^+^ lymphocyte proliferations and differentiations in patients that may subsequently develop into functional cytotoxic T (CD8^+^ T) cells, which suggests that CCL19 may help HBV clearance in AHB, HCC, and CHB individuals. Hence, to study the mechanisms and effects of promotion Ag-responsive immune activations by CCL19 in vivo, we further analyzed the immune responses and virus clearance in CCL19 over expression (CCL19 + HBV) and LV-CCR7 knockdown (shCCR7 + HBV) CHB mouse models.

### CCL19 over expression accelerates HBV clearance in murine livers

**Fig. 1:**
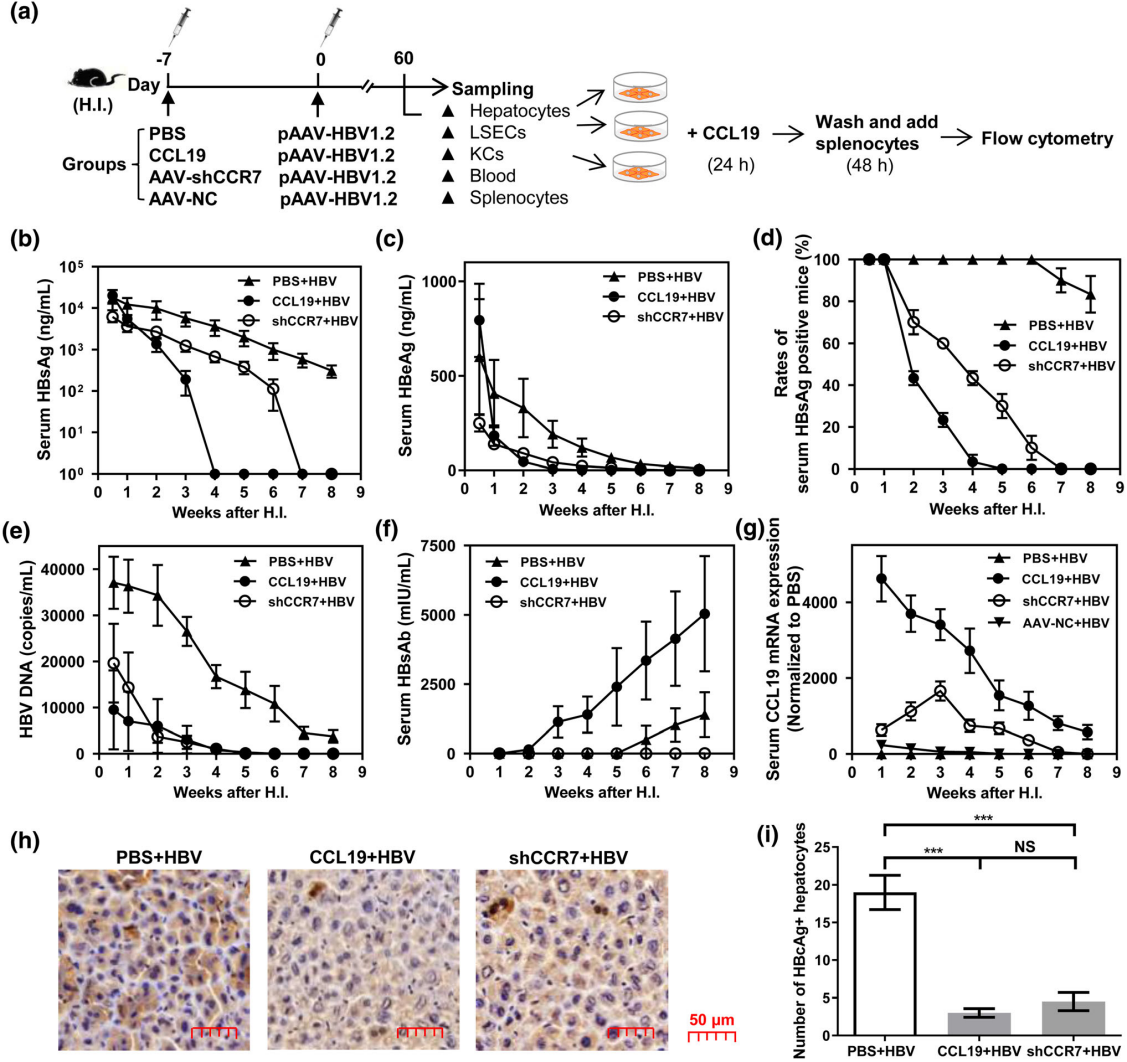
Pretreatment of mice with CCL19 plasmid promotes HBV clearance in vivo. **a** Establishment of CCL19 over expression, CCR7 knockdown and HBV chronic mouse models by H.I. and schedule. Levels of serum HBsAg (**b**), HBeAg (**c**) and HBsAb (**f**) were detected by ELISA. **d** The rates of serum HBsAg-positive mice in the three CHB mouse models. **e** Copies of intrahepatic HBV DNA in the three CHB mouse models detected by real-time PCR. **g** Expression of serum CCL19 mRNA in the three CHB mouse models detected by real-time RT-PCR. **h** Staining HBc-positive hepatocytes of three CHB mouse models at 4 wpi by IHC assay. Quantification of HBc^+^ cells in five high-power fields for each mouse. The data are presented as the means ± SEMs. **i** Comparison of the number of HBc-positive hepatocytes. Five to seven mice were analyzed per group, and at least two independent experiments were performed. Significant differences between the groups are indicated: ***p < 0.001, *NS* not significant

**Fig. 2:**
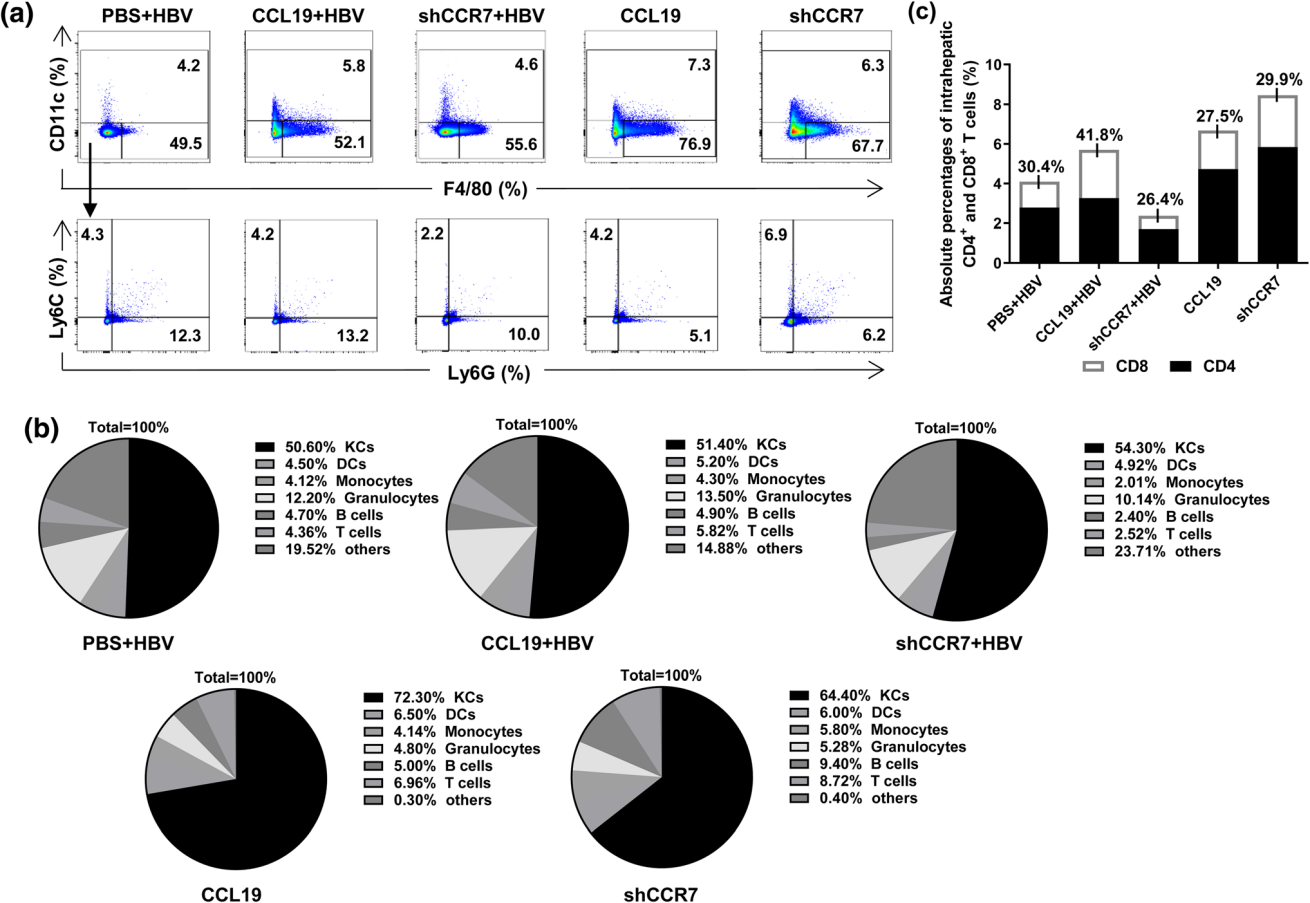
CCL19 mediates intrahepatic CD8^+^ T-cell increase in vivo. At 2 wpi, intrahepatic NPCs were isolated and analyzed by flow cytometry. **a** Frequencies of KCs (F4/80^+^, CD11c^−^) from CHB mouse models and naïve control. **b** Comparison of the compositions of NPCs in livers. **c** Comparison of absolute percentages of CD4^+^ and CD8^+^ T cells in livers. Five mice were analyzed per group, and at least two independent experiments were performed

It has been shown to enhance resistance to various viral infections in murine models by allowing CCR7^+^ T cells and dendritic cell to migrate to the spleen and lymph nodes [26, 43]. High persistence rate of HBV has been established in a hydrodynamic injection-based transfection model in C57BL/6 mice [30]. To examine the effects of CCL19 on HBV replication or clearance, a week in advance, C57BL/6 mice were first treated with recombinant murine CCL19 plasmid (10 μg per mouse), AAV-shCCR7 (1.92 × 10^10^ vg/ml per mouse) to knockdown CCR7 expression and the same volume PBS by H.I., after which pAAV-HBV1.2 plasmid was introduced into C57BL/6 mice by H.I. at day 0 to establish HBV chronic infected mice model (Fig. 1a). Because HBc Ag is crucial for the HBV life cycle and affects HBV persistence rates, we examined intrahepatic viral replication, transcription and HBc Ag protein expression in murine liver. The serum levels of HBsAg and HBeAg were assessed by specific ELISA. The results showed that levels of HBsAg (Fig. 1b), HBeAg (Fig. 1c), the rates of serum HBsAg-positive mice (Fig. 1d) and the copies of intrahepatic HBV DNA (Fig. 1e) were significantly decreased in the CCL19 + HBV and shCCR7 + HBV groups, and the outcomes of antigen and viral DNA clearance were similar of these two groups. The level of serum HBsAb significantly increased in the CCL19 + HBV group at 2 wpi (Fig. 2f). In contrast, CCL19 mRNA expression was significantly higher not only in the CCL19 + HBV group, and but also in the shCCR7 + HBV group at 2 wpi, while its expression was not observed in the AAV-NC + HBV, sole CCL19 stimulation and shCCR7 groups (Fig. 1g). The number of HBc-positive hepatocytes was significantly decreased in the CCL19 + HBV and shCCR7 + HBV groups (Fig. 1h, i). Similarly with intrahepatic HBV DNA decrease, the amounts of HBV DNA in sera significantly decreased in the CCL19 + HBV and shCCR7 + HBV groups at 2 wpi (data not shown). Thus, CCL19 over expression could promote HBV clearance and increase the levels of protective Abs (anti-HBs and anti-HBc). Interestingly, we also observed HBV removal in the CCR7 knockdown mice, which may be associated with a delayed increase in CCL19 expression in vivo.

### CCL19 mediates increased numbers of intrahepatic CD8^+^ T cell in vivo

Except for hepatocytes, KCs and LSECs as hepatic NPCs are considered to be the most important and abundant Ag-presenting cells (APCs) in the liver, they can take up and present Ags to T cells and play a crucial role in promoting immune tolerance in the liver [44, 45]. These APCs are known to play an important role in the suppression of IFN-γ production and T-cell proliferation, which is attributed to KCs accounting for a large proportion of NPCs (~ 58%) [29]. For the initial assessment effects of CCL19 over expression and its ability to reverse the suppressive properties of hepatocytes and NPCs to induce T-cell immunity and promote intrahepatic HBV clearance, we first analyzed the composition of NPCs in CHB, CCL19 over expressed and its receptor CCR7 decreasing expressed CHB mice at 2 wpi. Compared to our previous study [29], CCL19 high expressed mice showed a significantly higher frequency (~ 70%) of KCs (F4/80^+^, CD11c^−^) in total NPCs, and knockdown of CCR7 led to a partial reduction in the proportion (~ 60%) of KCs, and the differences of frequencies were not apparent in CHB mouse groups (~ 50%) (Fig. 2a). However, HBV infection resulted in reductions of KC (CD45^−^, IA/IE^+^, F4/80^+^) and T cell (CD3^+^, CD45^+^) percentages and an increase of granulocyte (CD45^+^, CD11c^−^ and Ly6G^+^) percentage compared with mice injected with CCL19 plasmid or AAV-shCCR7 alone in hepatocytes (Fig. 2a–c, supp. Fig. 2a). A lower proportion of KCs in CHB mice was observed than previously reported [29]. Compared with that observed in the PBS + HBV and CCL19 + HBV groups, shCCR7 + HBV mice developed obvious decreasing levels of monocytes, T cells, B cells and granulocytes in liver, (Fig. 2b). We further assessed whether the clearance of HBV mediated by CCL19 was due to increased T-cell subclass proliferation in vivo. By comparing the percentage compositions of CD4^+^ and CD8^+^ T cells by flow cytometry, we observed that HBV infection could decrease the percentage of T cell, and CCL19 could help to increase the proportion of T cells, including both subclasses in CHB mice at 2 wpi. More importantly, the increase in the absolute percentage of CD8^+^ T cells was obvious (41.8%) in CCL19 over expression group, and knocking down CCR7 in CHB mice developed an obvious decrease in T cells and subclasses (Fig. 2c). Therefore, our results indicate that CCL19 could promote expansion of the proportion of intrahepatic CD8^+^ T cells, which maybe an important reason for the observed HBV immune clearance by cellular immune responses. However, the reason for the observed HBV clearance in the CCR7 knockdown group remains to be further elucidated.

### CCL19 mediates PD-1^+^ CD8^+^ T-cell decrease in blood during viral clearance

**Fig. 3:**
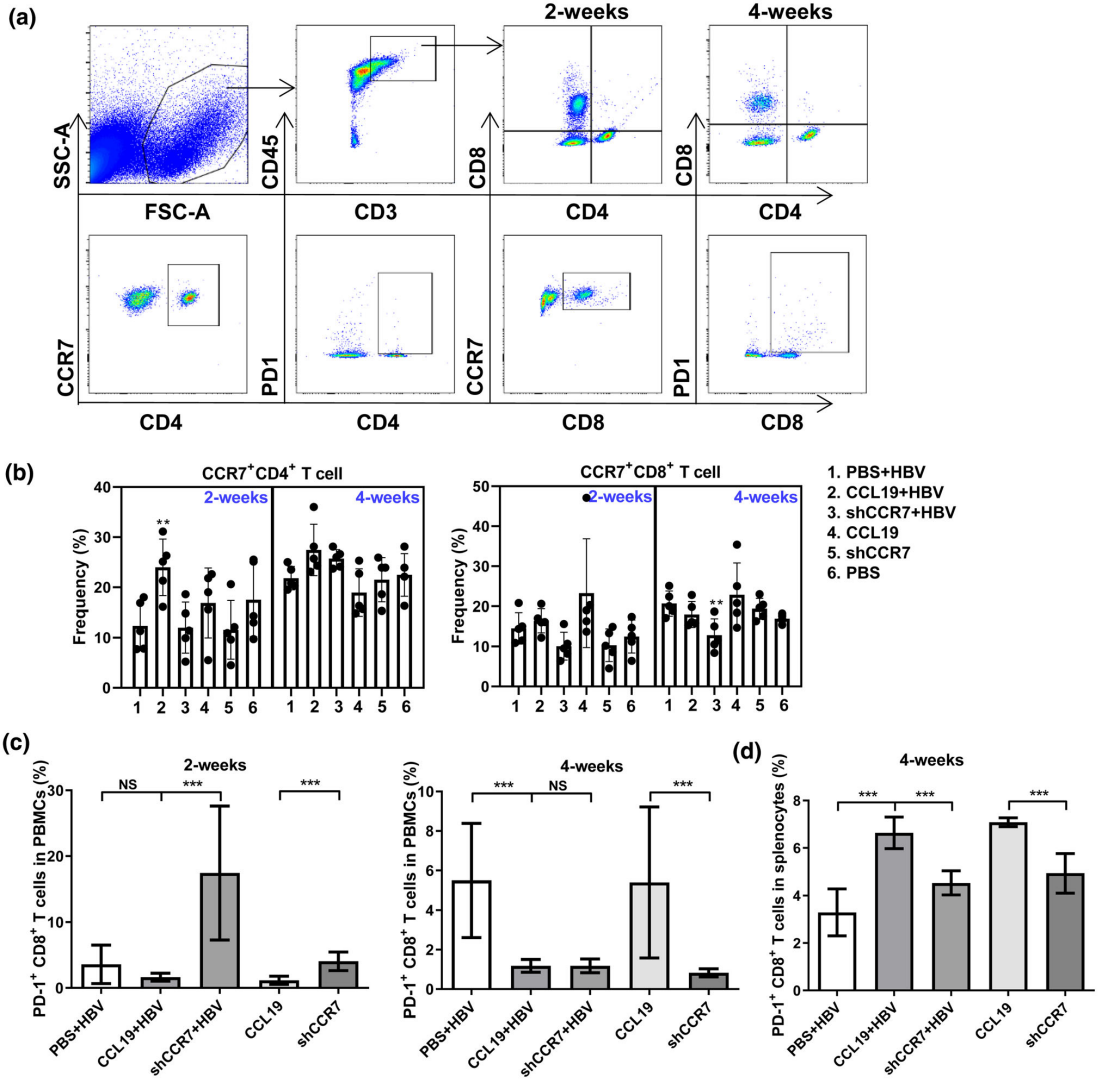
CCL19 over expression mediates decreased numbers of PD-1^+^ CD8^+^ T cells in blood and increased numbers in spleens. Freshly isolated PBMCs at 2 and 4 wpi were evaluated for the frequencies of CCR7^+^ and PD-1^+^ on CD4^+^ or CD8^+^ T-cell surfaces by flow cytometry, respectively. **a** Graph of CCR7 analysis by flow cytometry. **b** Frequency of CCR7 expression on CD4^+^ and CD8^+^ T cells at 2 and 4 wpi. **c** Frequency of PD-1^+^ CD8^+^ T cells in blood at 2 and 4 wpi. **d** Frequency of PD-1^+^ CD8^+^ T cells in spleens at 4 wpi. Five to seven mice were analyzed per group, and at least two independent experiments were performed. The data are presented as the means ± SEMs in panels. Significant differences between the groups are indicated: **p < 0.01; ***p < 0.001; *NS* not significant

The migration of T cells to lymph nodes is regulated by chemokine-receptor CCR7 in response to CCL19 [26]. As CCR7 is expressed in naïve and central memory T cells, CCR7 deficiency may impair T-cell functions and viral control [26, 46]. The production of Abs mediated by Th1 cells is known to depend on the CCR7 and its ligand CCL19 [31, 47], and because significant Ab response (HBs Ab) was observed in our study when CCL19 was used to pretreated mice (Fig. 1f), we further examined whether the CCL19-induced increase in HBV-specific Abs correlated with an increased number of CCR7^+^ CD4^+^ T cells. Flow cytometry analysis of PBMCs and splenocytes revealed that external CCL19 stimulation promoted significantly higher levels of CCR7^+^ CD4^+^ T cells in blood at 2 wpi, while knocking down CCR7 mice resulted in significantly lower levels of CCR7^+^ CD8^+^ T during HBV infection up to 4 wpi (Fig. 3a, b).

**Fig. 4:**
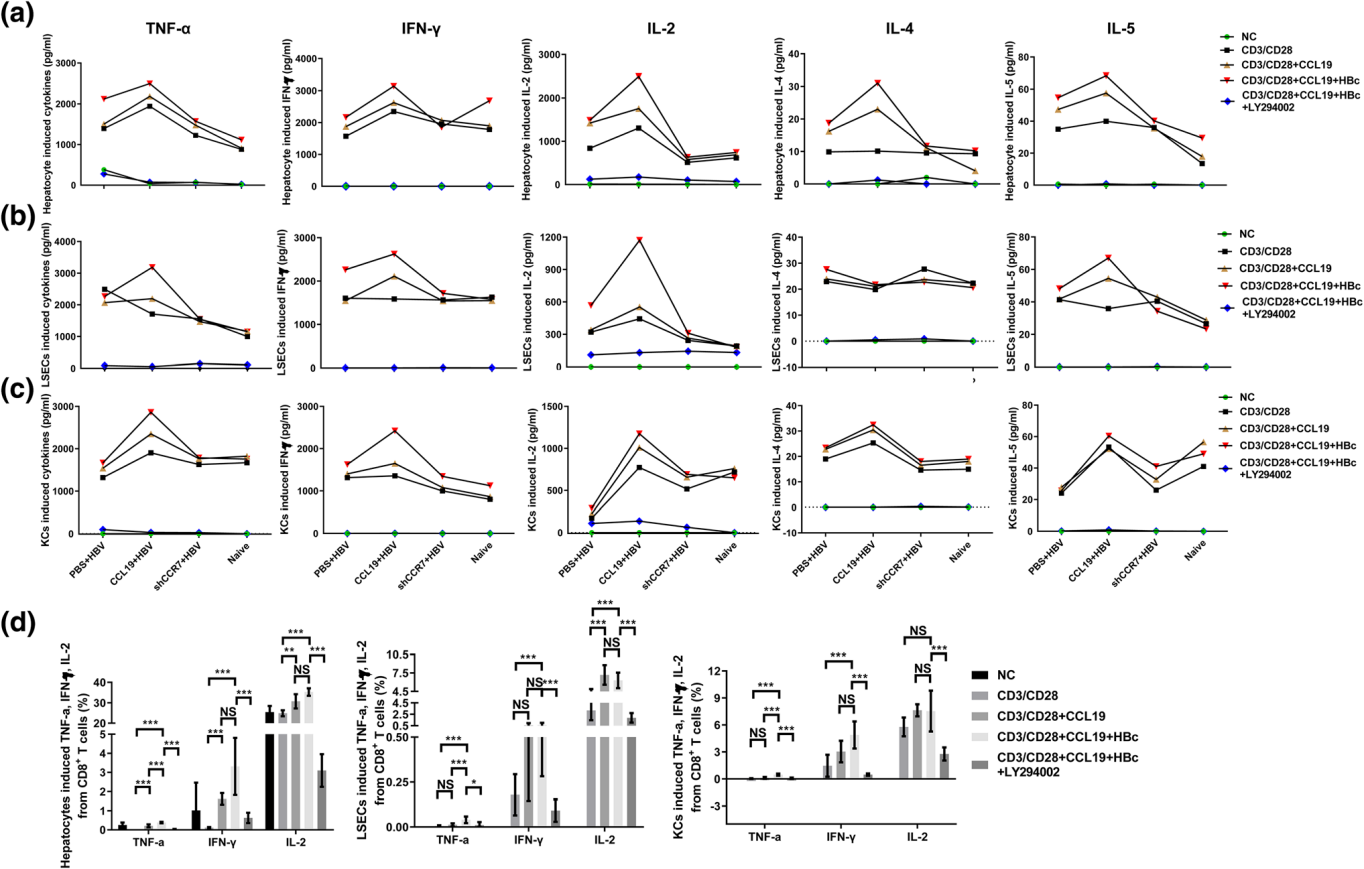
CCL19 mediates reversing the suppressive properties of hepatocytes and NPCs to induce T-cell immunity and secretive cytokines. C57BL/6 mice were injected with PBS, CCL19 and shCCR7 plus pAAV-HBV1.2, separately. After 60 dpi, hepatic APCs (hepatocytes, CD146^+^ LSECs and F4/80^+^ KCs) were separated by centrifugation and MACS sorting and then treated with or without 100 ng/ml murine CCL19 protein stimulated hepatocytes, LSECs or KCs were incubated 24 h. Then, after discarding the supernatants and washing the cells, fresh splenocytes from experimental gourps stimulated with anti-CD3 (1 μg/ml) and anti-CD28 (1 μg/ml) were added (2 × 10^6^/well), and the cells were co-cultured at a ratio of 1:2 (APCs: splenocytes). The supernatants from co-cultivation for 48 h with hepatocytes (**a**), LSECs (**b**) and KCs (**c**) were analyzed by flow cytometry for the Ag-responsive TNF-α, IFN-γ, IL-2, IL-4 and IL-5. **d** Intracellular cytokines of CD8^+^ T cells involved in TNF-α, IFN-γ, and IL-2 production were assessed by flow cytometry in CCL19 + HBV group. The data are shown as superimposed symbols at the mean with connecting lines of one out of two representative experiments, which were performed in triplicate wells. Five mice were analyzed per group, and at least two independent experiments were performed. The data are presented as the means ± SEMs in panels. Significant differences between the groups are indicated: *p < 0.05; **p < 0.01; ***p < 0.001; *NS* not significant

With the removal of HBV by specific and nonspecific lymphocytes, the number of viral-specific effector T lymphocytes will largely decrease due to apoptosis mediated by programmed cell death protein 1 (PD-1), after which a functional memory T-cell population or chronic infection is established [48]. PD-1 has been shown to be transiently upregulated by HBV-specific CD8^+^ T cells at the early phase of acute HBV infection, and dynamic decreases in PD-1 correlate with HBV-specific memory CD8^+^ T-cell development in acute self-limited hepatitis B patients [49, 50]. As shown in Fig. 4c, the number of PD-1^+^ CD8^**+**^ T cells decreased at 2 and 4 wpi in the CCL19 over expressed mice during HBV clearance, but displayed transient and significantly increases in the CCR7 knockdown mice at 2 wpi, thereafter decreasing with viral clearance (Fig. 3c). Consistent with the Fig. 1g, the CCL19 in CCR7 knockdown mice has increased since 2 wpi and up to 4 wpi. Similarly, the number of PD-1^+^ CD8^**+**^ T cells in blood was significantly decreased in the shCCR7 + HBV group at 4 wpi (Fig. 3c). A significantly high frequency of PD-1^+^ CD8^**+**^ T cells was observed in CCL19 high expressed murine splenocytes at 4 wpi (Fig. 3d). It's worth noting that the frequency of PD-1^+^ CD8^**+**^ T cells observed in naïve murine splenocytes (16.1 ± 1.3%) was apparently higher than that observed in experimental groups at 4 wpi (data not shown on graph). Taken together, our results indicate that CCL19 induces Ag-specific CD8^**+**^ T-cell apoptosis during HBV clearance, given that higher CCL19 expression in CHB mice could promote the accumulation of more Ag-specific PD-1^+^ CD8^**+**^ T cells in spleens that may be immediately recalled after Ag restimulation. This effect was only partially decreased in shCCR7 + HBV mice compared with that observed in the CCL19 + HBV mice, which may be associated with CCL19 delayed increase and maintaining a lower concentration in shCCR7 + HBV mice.

### CCL19 promotes hepatic APC-mediated CD8^+^ T-cell activations

Because peripheral blood is not a complete substitute for the immune response of liver tissue, we further analyze the condition of different groups of liver cells to promote antiviral cytokines secretion of TNF-α, IFN-γ and IL-2. The liver is a tolerogenic organ harboring parenchymal cells and different nonparenchymal cell populations that can serve as APCs but are poorly efficient in effector T-cell priming, with a propensity to induce T-cell tolerance rather than T-cell activation [29]. However, due to the paucity of information regarding how CCL19 stimulation influences the immunological function of hepatic APCs, we investigated whether CCL19 over expression in vivo and in vitro could influence the function of hepatocytes and NPCs from C57BL/6 mice and elucidated the mechanisms involved in parenchymal cell- and nonparenchymal cell-induced T-cell immunity. We assessed cellular immunity by coculturing CCL19-stimulated APCs (hepatocytes, LSECs and KCs) from experimental CHB mice with respective experimental murine splenocytes primed with or without HBc peptide, and then evaluated the Ag-responsive production of Th1/Th2-like cytokines in the supernatants of co-cultured cells and intrasplenocytes. As shown in Fig. 4a–c, CHB mice prestimulated with CCL19 could potently promote APC-mediated Ag-specific T-cell activation and was followed by the production of immune regulatory cytokines, such as the Ag-responsive Th1-like cytokines TNF-α, IFN-γ, IL-2, and Th2-like IL-4, IL-5, which was mediated by hepatocytes, LSECs and KCs. These responses were significantly weakened in CCR7 knockdown mice and nearly complete abrogated by LY294002. The levels of TNF-α, IFN-γ and IL-2 produced by APCs-mediated CD8^+^ T cells were significant high than that without CCL19 high expressed mice (Fig. 4d). The quantities of secreted cytokines were ranked as: IL-2 > IFN-γ > TNF-α, while IFN-γ and IL-2 were dominant secretion in APCs-mediated CD8^+^ T cells in experimental groups, with them producing a significantly high level of Ag-responsive TNF-α and IFN-γ (Fig. 4d). Taken together, CCL19 abrogated the suppression of hepatic APCs to promote CD8^+^ T-cell activation, and is an important mechanism during HBV clearance.

### CCL19 mediates the activation-induced cell death (AICD) of Ag-specific CD8^+^ T cells

**Fig. 5:**
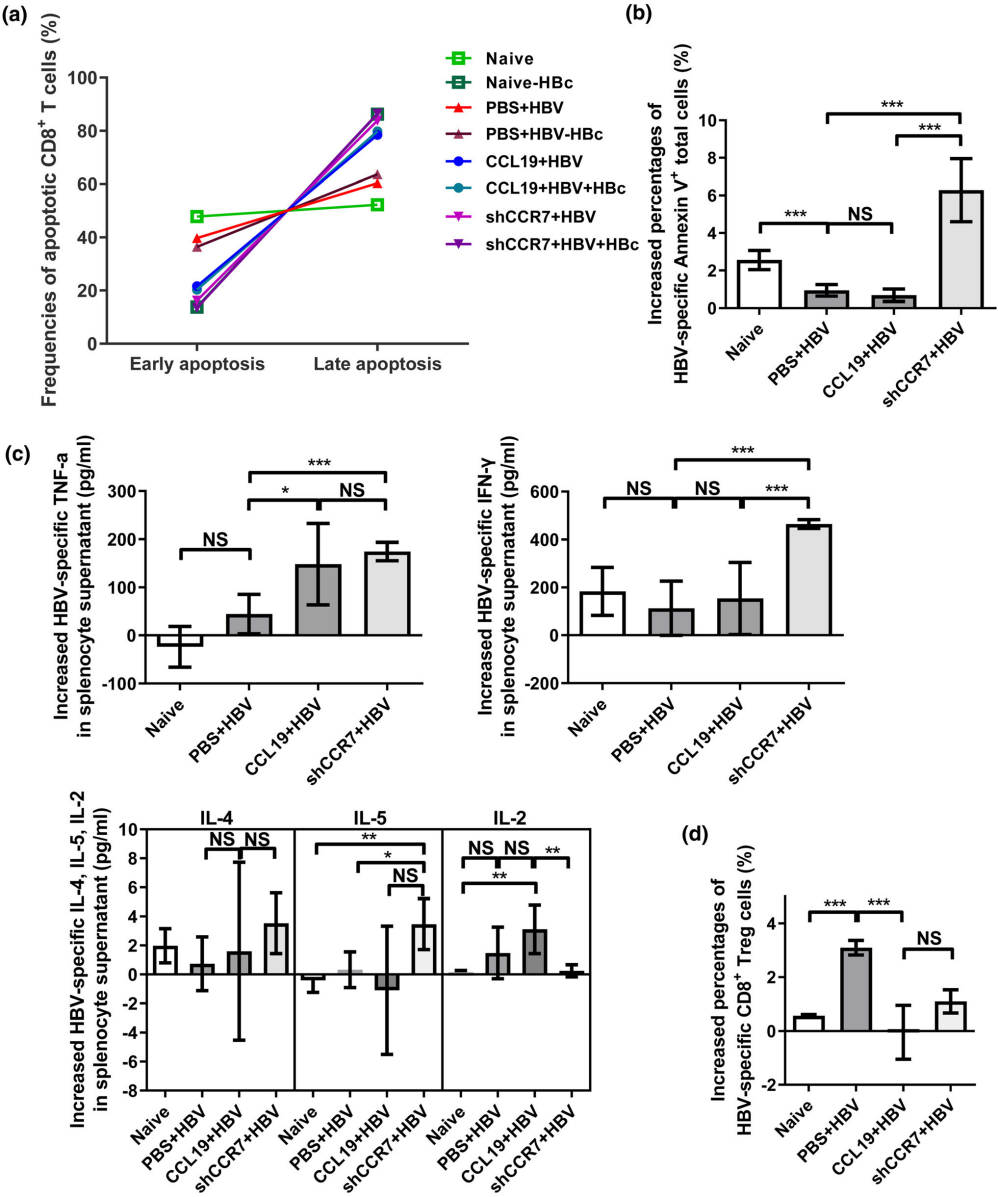
CCL19 mediates Ag-responsive CD8^+^ T cells apoptosis, T_reg_ cells and secretive cytokines. Freshly isolated splenocytes of the three CHB mouse models were collected at 60 dpi, stimulated with anti-CD3 plus anti-CD28 and HBc peptide in vitro, and then cultured for 48 h at 37 °C in an incubator under an atmosphere with 5% CO_2_. Annexin V^+^/PI^+^ CD8^+^ T cells were detected by flow cytometry. Early apoptosis: FITC-Annexin V (+) and BV510-PI (−); late apoptosis: FITC-Annexin V (+) and BV510-PI (+). **a** Detection of the apoptotic lymphocytes (CD8^+^ T cells) after HBc peptide stimulation in vitro. **b** Increased percentages of HBV-induced late apoptotic (Annexin V^+^) total cells (CD4^+^ plus CD8^+^ T cells). **c** Increased levels of cytokines in the supernatants of splenocytes were assessed by flow cytometry after with and without HBc peptide stimulation in vitro. **d** Increased CD8^+^ T_reg_ cells were detected by flow cytometry after with and without HBc peptide stimulation. The data are presented as the means ± SEMs of one out of two representative experiments, which were performed in triplicate wells. Increased percentage of apoptotic cells or T_reg_ cells (%) = (frequencies_(murine splenocytes + HBc)_—frequencies_(murine splenocytes)_)/frequencies_(murine splenocytes)_ × 100 (%); Increased cytokines = doses _(murine splenocytes + HBc)_—doses_(murine splenocytes)_. Five mice were analyzed per group, and at least two independent experiments were performed. Significant differences between the groups are indicated: *p < 0.05; **p< 0.01; ***p < 0.001; *NS* not significant

Apoptosis is a type of programmed cell death that regulates cellular homeostasis by removing damaged or unnecessary cells, thereby blunting the host immune response to many viruses that evade, obstruct, or subvert apoptosis, which is subsequently accompanied by viral clearance [51, 52]. In a previous study, in vitro stimulation of CD4^+^ T cells with CCL19 and anti-CD3 plus anti-CD28 was shown to develop significantly enhanced AICD [53]. In addition, a signal-blocking molecular mechanism shows that blocking downstream CCR7 signal adaptors (PI3K/AKT) can also induce apoptosis [26, 51, 52], we speculate that knockdown signaling molecules will also have this effect. To further elucidate the underlying mechanism of HBV immune clearance after CCL19 over expression and CCR7 knockdown in vivo, we further investigated the potential mechanisms underlying cellular immunity. Our results showed that CD8^+^ T cells from both CCL19 over expressed and CCR7 knockdown CHB mice developed high frequency of late apoptotic cells (Annexin V^+^ and PI^+^) after HBc peptide and anti-CD3 plus anti-CD28 stimulation compared with PBS + HBV group (Fig. 5a). Interestingly, shCCR7 + HBV group developed a significantly high level of apoptotic total T lymphocytes (Annexin V^+^ and CD3^+^ CD45^+^) after HBc stimulation, which was also observed in naïve mice (Fig. 5b). After HBc stimulation, splenocytes of CCL19 + HBV mice only produced higher levels of Th1-like cytokines, such as TNF-α, while shCCR7 + HBV mice produced a significantly high levels of antiviral cytokine TNF-α, IFN-γ and IL-5, compared to PBS + HBV group (Fig. 5c). The numbers of Ag-responsive CD8^+^ T_reg_ (CD25^+^ FoxP3^−^) cells were significantly decreased in the CCL19 + HBV and shCCR7 + HBV groups compared with that observed in the PBS + HBV group (Fig. 5d). Taken together, these results indicate that overexpressed CCL19 and knocking down CCR7 promote the AICD of Ag-responsive CD8^+^ T cells, which means the generation of more activated T cells, rather than inhibition (T_reg_ cells). Knocking down CCR7 promotes more lymphocytes to produce immune-activated apoptosis (Annexin V^+^ total cells), and it does not affect the Ag-responsive antiviral cytokine IFN-γ production in vitro*.* Therefore, it is suggested that disrupting the balance of the CCL19/CCR7 axis may be an important mechanism for HBV immune clearance.

## Discussion

It is generally known that HBV replication is noncytopathic, and does not induce a measurable innate immune response in the infected liver, and the outcome of infection is determined by the kinetics, breadth, vigor, trafficking, and effector functions of HBV-specific adaptive T-cell responses and the development of neutralizing antibodies [42]. From HBV patients’ in vitro experiments, we found that CCL19 protein enhanced the frequencies of Ag-responsive IFN-γ^+^ CD8^+^ T cells from patients by approximately twofold, while CCR7 knockdown partially suppressed IFN-γ secretion. In supp. Table 1, ALT or HBV DNA indicates the inflammation or removal of virus, respectively. If IFN-γ increases, it will help to clear the virus and reduce the inflammation in hepatitis B patients. Dysregulation of one or more of these events leads to persistent HBV infection and a variably severe chronic necroinflammatory liver disease that fosters the development of HCC. However, little is known regarding the factors modulating intrahepatic CTL function or the complex interactions in the liver microenvironment that lead to liver immunopathology. In the present study, for the first time, the role of CCL19 in mediating Ag-responsive CD8^+^ T-cell activation and promoting viral clearance in a mouse model were elucidated.

Consistent with the results of our previous studies, CCL19 co-immunized with Ag promotes Ag-specific IFN-γ secretion by murine splenocytes after Ag restimulation in vitro [35]. In CHB patients, we demonstrated that CCL19 can rescue HBV-responsive or non-responsive (without HBc peptide) dysfunctional CD8^+^ T cells and produce antiviral cytokine IFN-γ in vitro. Furthermore, when CCR7 receptor was downregulated or blocked on PBMCs, IFN-γ expression was significantly decreased in supernatants (Supp. Fig. 1). According to the results of Supp. Fi. 1a and 1b, CCL19 alone is more effective to promote IFN-γ secretion, that is, if there is no activation of HBV (HBc expression), its effect is more obvious. If there is activation of HBV (without HBc expression), although this effect is weakened in partial patients, it is still meaningful (supp. Fig. 1c–e). Hence, CCL19 has application prospects as an immunomodulator to help HBV clearance. Its role is more complicated in vivo, and further research is needed. In mouse models, we also observed that CCL19 could accelerate HBV clearance with increased protective Abs. Notably, we observed delayed CCL19 expression in the sera of CCR7 knockdown mice, which resulted in a weaker immune response in HBV infection and subsequent viral clearance, the mechanism for which remains to be further elucidated. We speculate that it may be that CCL19 is in charge of immune-homeostasis, so it will increase secretion to maintain immune function in CCR7 knockdown mice. Hence, CCL19 may help to produce Ag-specific protective Abs (HBs Ab), which contribute to eliminate circulating viral particles and infected cells by humoral immune responses.

**Fig. 6:**
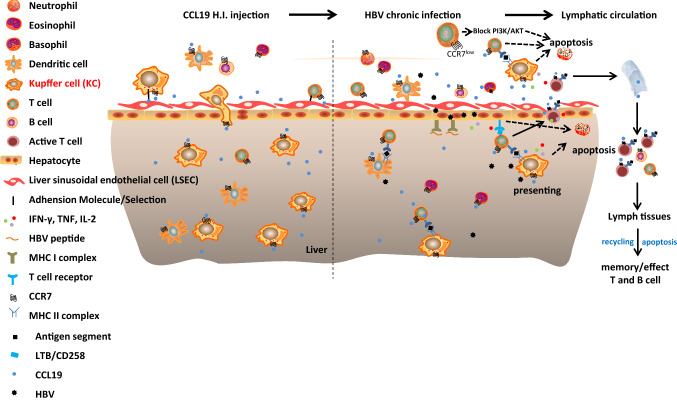
Schematic model of the mechanism by which CCL19 promotes KC migration into liver and AICD after HBV infection (apoptosis) in vivo. CCL19 over expression in vivo directs the migration of CCR7^+^ macrophages, DCs and T cells to penetrate hepatic vascular bed and settle in the liver. Ag-responsive CD8^+^ T cells apoptosis, After HBV infection, CCL19-stimulated NPCs mediate T-cell activation and promote apoptosis during viral clearance. Knockdown of CCR7 expression downregulates the phosphorylation of PI3K/AKT, resulting in the tendency of CCR7-expressing cells tend to undergo apoptosis

It is generally accepted that infection with HBV and HCV triggers the activation of immunological events involving virus-specific cytotoxic T lymphocytes (CTLs), which aids in the control of viral replication and, at the same time, causes liver inflammation and damage due to the recruitment of Ag-nonspecific inflammatory cells [39]. However, additional inflammatory cells were not observed distributed within the liver in CCL19 over expressed or CCR7 knockdown CHB mice compared with the PBS + HBV mice at 2 wpi accompanied with HBsAg decrease (Fig. 1d). In our present study, CCL19 contributed to stimulating a high frequency of KCs and CD8^+^ T cells in liver (Figs. 2b, c, 6), decreasing the proportion of PD-1-expressed CD8^+^ T cells (Fig. 3c) and Ag-responsive CD8^+^ T_reg_ cells which circulate in blood (Fig. 5b). Hence, it suggests that CCL19 promotes the proliferation and differentiation of CD8^+^ T cells in liver post HBV infection and downregulates Ag-responsive CD8^+^ T_reg_ cells, which may contribute to eliminate intrahepatic and circulating HBV particles, or infected cells by cellular immune responses.

Previous studies have shown that activated murine NPCs, KCs and LSECs can suppress HBV replication [36] and that LSECs are fully efficient APCs [45]. The goal of our present study was to elucidate the role and mechanism of CCL19 in suppressing HBV replication and promoting HBV clearance. We observed that CCL19 pretreatment results in murine hepatic APC activation and could mediate CD8^+^ T-cell activation and promote Th1/Th2-like cytokine production (Fig. 6), which was partially abolished in CCR7-downregulated mice (Fig. 4a–d). Antibody responses depend on the function of Th1 cells, which may also be the primary reason for production of potent Ag-specific Abs in the CCL19 over expression groups. However, the mechanism of HBV clearance in the CCR7 knockdown group is may be different or associated with CCL19 delayed expression. LSECs play an important role in cross presentation of Ags from virus-infected hepatocytes and promote effector CTLs to release TNF-α, which kills virus-infected hepatocytes through caspase activation [54]. A previous study also showed that TNF-α plays an important role in the inhibition of HBV reactivation [55]. The results of our present study showed that in vitro, HBc peptide could be used to restimulate hepatocytes, LSECs and KCs, and the Ag peptide was cross-presented followed by the release of notably high levels of TNF-α, IFN-γ, IL-2, IL-4 and IL-5 in supernatants. However, increased TNF-α may play a crucial role in enhancing activation-induced apoptosis in HBV-infected cells in CCL19 over expressed CHB mice. IFN-γ and IL-2 displayed high expression in the supernatants of co-cultured cells and intra CD8^+^ T cells, which are important to eliminate infected cells under APCs-mediating.

CCL19 is known to mediate T-cell trafficking, but little is known regarding its role as functional molecule in mediating HBV clearance. A paucity of lymph node T cells was observed in mutant mice lacking CCL19 expression that were primed with Ag, and the proliferation and recall response of CD4^+^ T cells was prolonged compared with the responses observed in wild-type mice [53]. The data showed that the Ag-stimulated apoptotic cell frequencies only continuously increased in wild-type mice and never increased in mice lacking CCL19 during the clonal contraction phase [53]. In our mouse model, both the CCL19 + HBV and shCCR7 + HBV groups displayed increased HBV clearance (Fig. 1d). Interestingly, mice in the shCCR7 + HBV group also exhibited increased CCL19 expression in blood during viral clearance (Fig. 1g). However, there may be differences in the viral removal and immune activation mechanisms of the two groups. In addition, our investigations showed that virus can mimic the function of CCR7 and promote viral infection and migration infection [26]. At beginning of the shCCR7 + HBV group, HBsAg and HBeAg were less than the CCL19 + HBV mice (Fig. 1b, c), the reason may be that CCL19 decreased production in CCR7 knockdown mice, or there are other mechanisms. For example, the CCR7 mAb that inhibits the function of CCL19 was added alone in the patients’ PBMCs in vitro, while accompanied with the secretion of IFN-γ increased instead at 72 h (Supp. Fig. 1b), and this mechanism needs further study. During infection, the clearance rates of pathogens is gradually accelerated, and the outcome is similar to that of the CCL19 + HBV group, which may be related to the compensatory production of CCL19 in the tissues, and mediating immune activation. Finally, CCL19 promotes the activation of immune cells and eliminates pathogens.

A similar phenomenon was also observed in a mutant mouse model lacking CCL19 expression, which ultimately displayed enhanced T-cell responses that persisted for a long time, which never occurs in wild-type mice [56, 57]. Therefore, the lack of CCR7 expression may be similar to this situation, which will reduce the number of lymphocytes that migrate to secondary lymphoid tissues. Thus, regarding the observed CCR7 downregulation mediates enhancement of T-cell responses, one possibility might be that HBV-activated T cells may stay in the draining lymph nodes in CCR7-downregulated mice much longer than in control mice due to little competition for a survival niche. Another possible explanation is that the delayed expressed CCL19 may be involved in priming and preparing CD4^+^ T cells for the regulation of their response to the subsequent stimulation. Most importantly, the block in CCR7 signaling has been reported to be involved in abrogating the suppression of apoptosis by the PI3K signal pathway and promote clearance of Ag-specific T cells after their activation [26, 51, 52]. In the present study, we analyzed the AICD of Ag-activated CD4^+^ (data not shown) and CD8^+^ T cells from H.I. mouse models. We observed that the frequency of Ag-responsive apoptotic CD8^+^ T cell was increased in mice pretreated with CCL19 plasmid and shCCR7. Our results revealed the mechanisms by which CCL19 activates cellular immunity and maintains cellular homeostasis during HBV infection, which helps to elucidate T-cell fate after Ag activation other than T-cell migration. In summary, CCL19-mediated crosstalk between CCR7 and PI3K/Akt promotes HBV-responsive CD8^+^ T-cell proliferation and apoptosis, and may ultimately potentiate HBV elimination (Fig. 6). Therefore, dual targeting of both the CCR7 receptor and the PI3K signaling axes may be a potential therapeutic avenue to specifically inhibit the functions of chronic HBV.

## Supplementary Information

Below is the link to the electronic supplementary material.

Supplementary file1 (PDF 1073 KB)

## Abbreviations

CCL19: Chemokine (C–C motif) ligand 19
CCR7: C–C chemokine-receptor type 7
LV: Lentiviral vector
AAV: Adeno-associated virus
shRNA: Short hairpin RNA
NC: Negative control
HC: Healthy control
HBV: Hepatitis B virus
CHB: Chronic hepatitis B
AHB: Acute hepatitis B
HBeAg: Hepatitis B e antigen
HBsAg: Hepatitis B surface antigen
HBsAb: Hepatitis B surface antibody
H.I.: Hydrodynamic injection
PBS: Phosphate buffer solution
MACS: Magnetic activated cell separation
IHC: Immunohistochemical
TLRs: Toll-like receptors
HBc: Hepatitis B core antigen
T cell: T lymphocyte
B cell: B lymphocyte
PD-1: Programmed cell death protein 1
APC: Antigen-presenting cell
PBMC: Peripheral blood lymphocyte
DC: Dendritic cell
NPC: Nonparenchymal liver cell
iMDC: Immature myeloid-derived cell
KC: Kuffer cell
LSEC: Liver sinusoidal endothelial cell
T_reg_: Regulatory T cell
PI3K: Phosphatidylinositol 3-kinase phosphoinositide 3-kinase
IFN: Interferon
TNF: Tumor necrosis factors
IL: Interleukin
ALT: Glutamate transaminase
CBA: Cytometric bead array
