# Supplementary figures and images for: CCL19 enhances CD8+ T-cell responses and accelerates HBV clearance
Source: J Gastroenterol. 2021 Jul 3;56(8):769–85. doi: 10.1007/s00535-021-01799-8 (PMC8316179; doi:10.1007/s00535-021-01799-8)

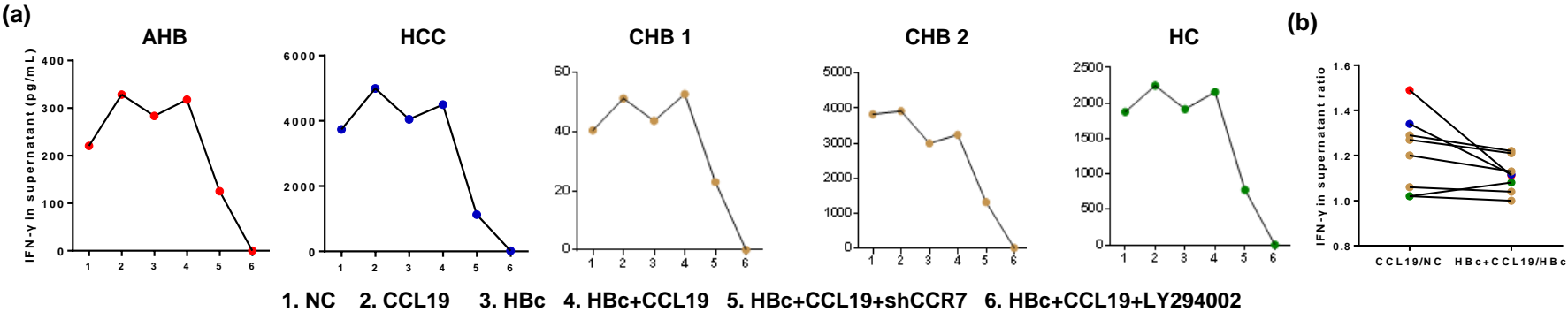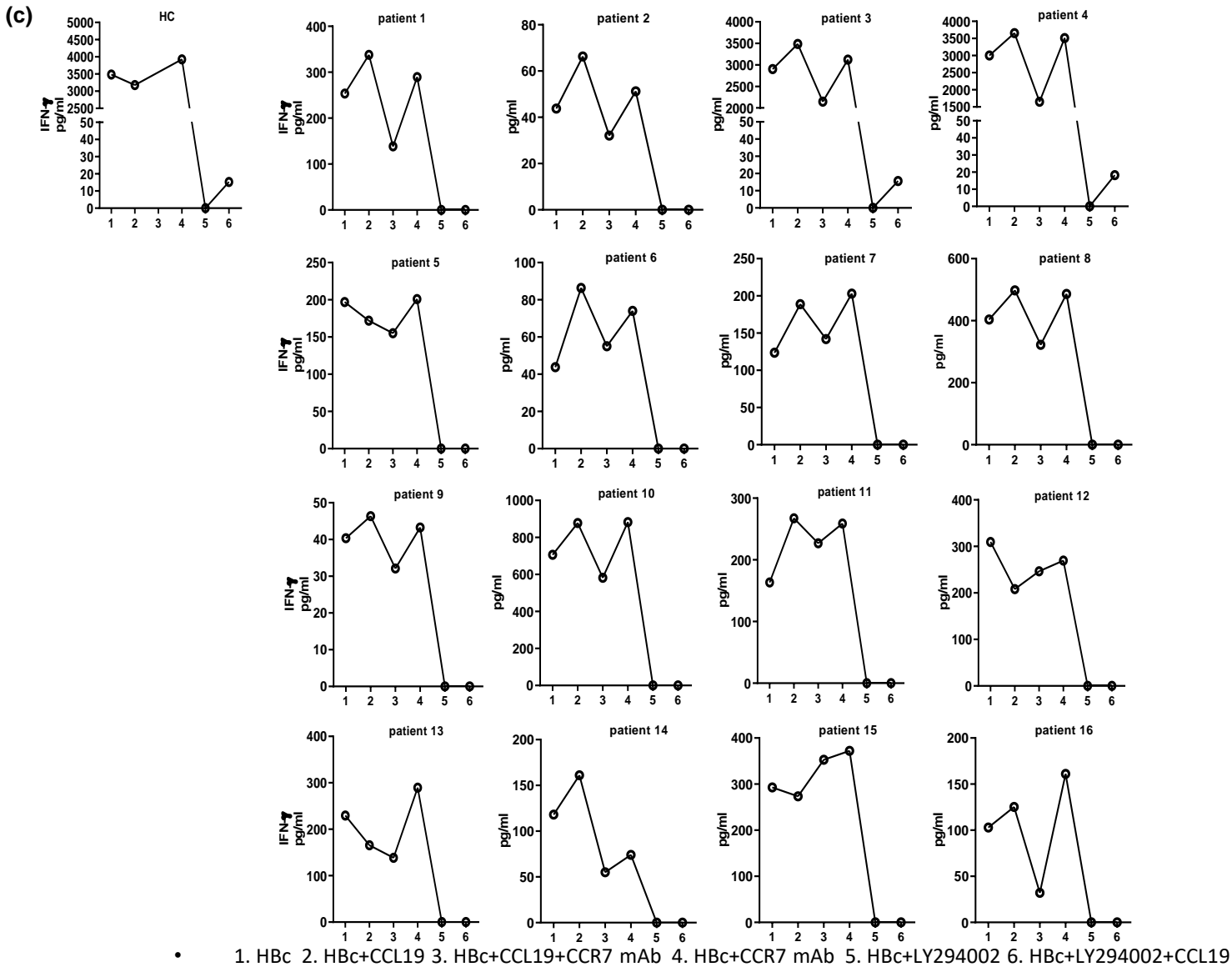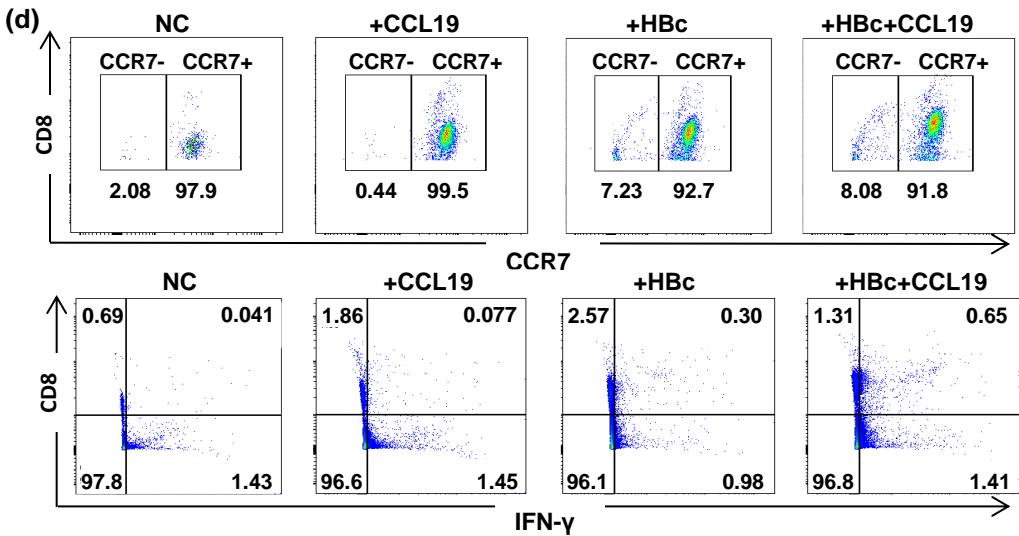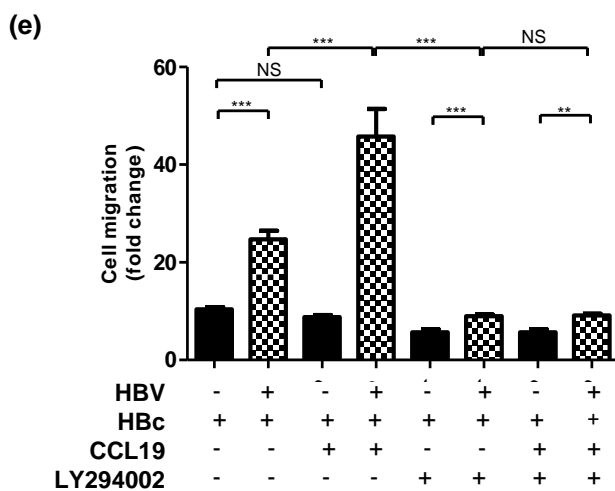

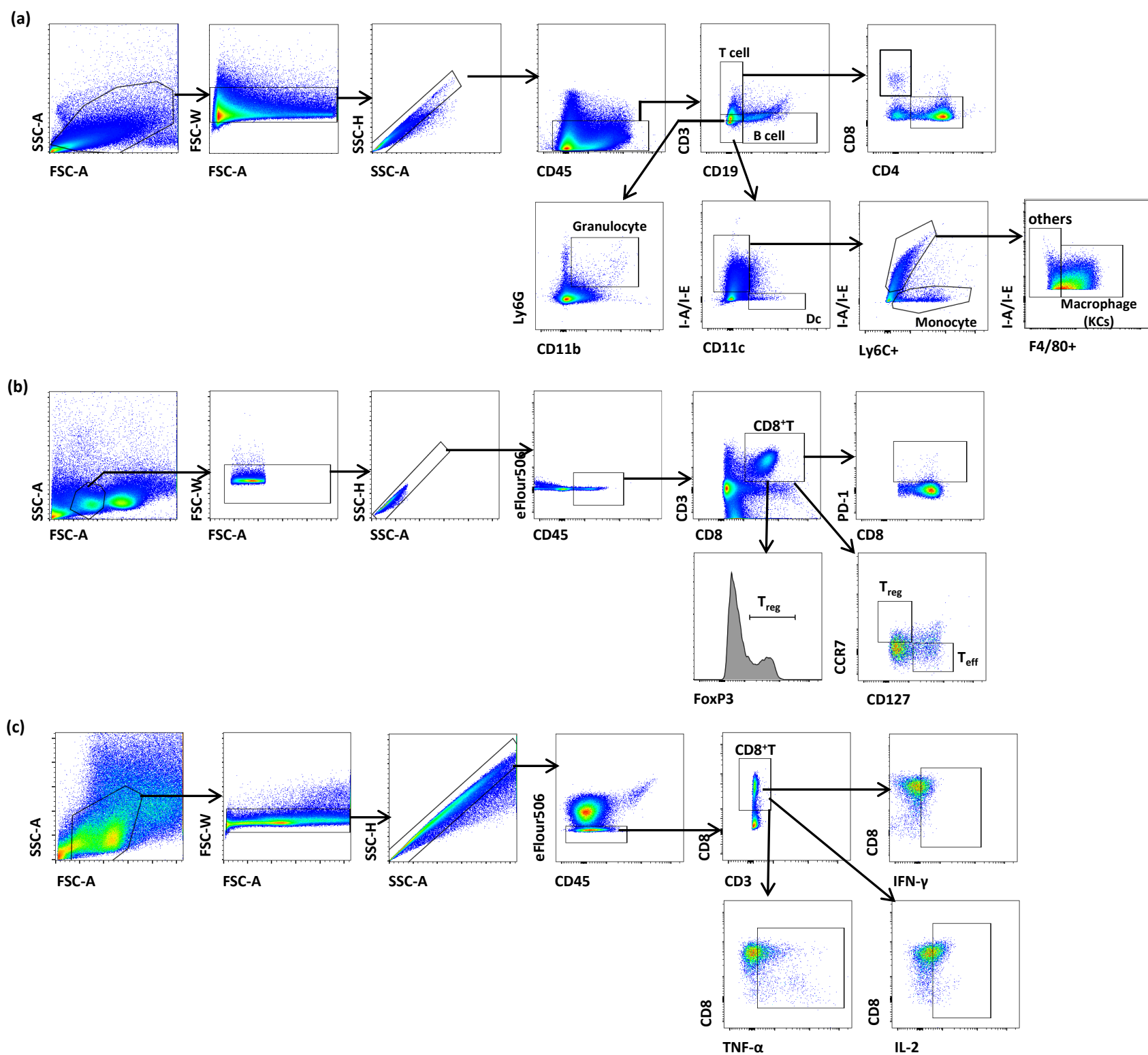

Supplement: Supplementary file 1 — Supplementary file1 (PDF 1073 KB) [file 535_2021_1799_MOESM1_ESM.pdf]
